# Inspiratory Muscle Training and Functional Capacity in Patients Undergoing Cardiac Surgery

**DOI:** 10.5935/1678-9741.20160035

**Published:** 2016

**Authors:** André Luiz Lisboa Cordeiro, Thiago Araújo de Melo, Daniela Neves, Julianne Luna, Mateus Souza Esquivel, André Raimundo França Guimarães, Daniel Lago Borges, Jefferson Petto

**Affiliations:** 1 Faculdade Nobre (FAN), Feira de Santana, BA, Brazil.; 2 Hospital Aliança, Salvador, BA, Brazil and Universidade Salvador (UNIFACS), Salvador, BA, Brazil.; 3 Research Group in Cardiovascular Physiotherapy, Salvador, BA, Brazil.; 4 Instituto Nobre de Cardiologia/Santa Casa de Misericórdia de Feira de Santana (INCARDIO), Feira de Santana, BA, Brazil.; 5 University Hospital da Universidade Federal do Maranhão (HUUFMA), São Luís, MA, Brazil.; 6 Escola Bahiana de Medicina e Saúde Pública (EBMSP), Salvador, BA, Brazil.

**Keywords:** Training, Muscle Strength, Cardiac Surgical Procedures

## Abstract

**Introduction:**

Cardiac surgery is a highly complex procedure which generates worsening of lung function and decreased inspiratory muscle strength. The inspiratory muscle training becomes effective for muscle strengthening and can improve functional capacity.

**Objective:**

To investigate the effect of inspiratory muscle training on functional capacity submaximal and inspiratory muscle strength in patients undergoing cardiac surgery.

**Methods:**

This is a clinical randomized controlled trial with patients undergoing cardiac surgery at Instituto Nobre de Cardiologia. Patients were divided into two groups: control group and training. Preoperatively, were assessed the maximum inspiratory pressure and the distance covered in a 6-minute walk test. From the third postoperative day, the control group was managed according to the routine of the unit while the training group underwent daily protocol of respiratory muscle training until the day of discharge.

**Results:**

50 patients, 27 (54%) males were included, with a mean age of 56.7±13.9 years. After the analysis, the training group had significant increase in maximum inspiratory pressure (69.5±14.9 *vs.* 83.1±19.1 cmH_2_O, *P*=0.0073) and 6-minute walk test (422.4±102.8 *vs.* 502.4±112.8 m, *P*=0.0031).

**Conclusion:**

We conclude that inspiratory muscle training was effective in improving functional capacity submaximal and inspiratory muscle strength in this sample of patients undergoing cardiac surgery.

**Table:** 

Abbreviations, acronyms & symbols	
2MST	= 2-minute step test
6MWT	= 6-minute walk test
CC	= Cardiac surgery
CG	= Control group
ICU	= Intensive care unit
MIP	= Maximum inspiratory pressure
TG	= Training group
TUG	= Timed up and go test

## INTRODUCTION

Cardiac surgery is a procedure performed in patients with cardiovascular disease. Surgical treatment remains the therapeutic option related to better survival of individuals with coronary heart disease, as well as in patients with valvular heart diseases.

After cardiac surgery, various complications that will require specific care, especially in the respiratory system, can be observed. These complications can lengthen the hospital stay of patients, causing increased hospital costs and becoming an important cause of morbidity and mortality^[1]^. Recent studies have shown that early mobilization, such as withdrawal of bed and walking, improves the functional status of the patient, reducing the length of hospital stay. The physical therapy should be applied daily in patients, thus ensuring their motor recovery^[2,3]^.

The surgical procedure entails reduced respiratory muscle strength. Aiming to reestablish it, some strategies should be used, such as respiratory muscle training, which aims to improve functional capacity, respiratory muscle strength and reduce imminent risks in adult patients undergoing cardiovascular surgery^[4]^.

Respiratory muscle strength training is one of the procedures followed, among several ones also used for respiratory therapy. The respiratory muscle strengthening promotes better efficacy in airway clearance, inspiratory pressure and maximal expiratory, and prevent fatigue of the respiratory muscles^[5]^.

In addition to respiratory changes in post-surgical patients, they develop reduced peripheral muscle strength which can reduce the functional capacity thereof. As a functional evaluation method is necessary the application of tests. The classic test is the 6-minute walk test (6MWT), where patients are instructed to walk as fast as they can without running, as recommended by the American Thoracic Society, in a distance of 30 meters, marked every 3 meters, with the turning point marked by a cone, and measured the total distance covered by the patient within six minutes^[6]^. It is a practical and low cost test that can be applied in different ages and body mass index and adequately reflects the physical capacity of patients to perform routine tasks^[7,8]^.

Veloso-Guedes et al.^[9]^ compared the walk test results in a hallway with 20 and 30 meters in patients on the list liver transplantation, concluding that an 20 meters runner can be used safely and effectively as an alternative to 30 meters for 6MWD. In addition, Carey et al.^[10]^ found that the 6MWD is inversely proportional to the severity of liver injury and a distance less than 250 meters increases the risk of death for patients on the waiting list for transplantation.

Therefore, the aim of this study was to evaluate the influence of inspiratory muscle training on functional capacity submaximal and inspiratory muscle strength in patients undergoing cardiac surgery.

## METHODS

This study is a randomized controlled trial, performed at the Inpatient Unit of the Instituto Nobre de Cardiologia/Santa Casa de Misericordia, Feira de Santana, Bahia, Brazil. The study enrolled patients aged above 18 years, of both genders and undergoing heart surgery (coronary artery bypass grafting, aortic valve and/or mitral, atrial septal defect correction), admitted to the Intensive Care Unit (ICU) in the period from February to August 2015.

Patients who did not understand the techniques used were excluded from the study, in addition to those who refused to participate, those who presented hemodynamic instability during evaluations or performance of inspiratory muscle training, and those who have complications and readmitted to the ICU. These parameters contraindicate muscle training. This study was approved by the Research Ethics Committee of the Faculdade Nobre in Feira de Santana/BA under the Protocol 796,580. All participants signed a written informed consent form.

The patients underwent the evaluation, collecting demographics and medical history. In all patients we performed 6MWT, where the individual was instructed to walk as quickly as possible without running for six minutes in a hallway 30 m. After this period was evaluated the maximum number of meters that the patient could walk. For safety criteria, all patients were evaluated for hemodynamic (blood pressure, heart rate and double product) and lung function (respiratory rate and oxygen saturation), before, during and after the walk test. If there was an increased heart rate and higher blood pressure to 20% of baseline value, the test was stopped. In addition, we evaluated the inspiratory muscle strength (maximum inspiratory pressure, MIP) via an analogue manometer. During the evaluation, a maximal expiration was requested to residual volume and then a maximum and slow inspiration to total lung capacity, and this test was performed using the method with the one-way valve. The patient repeated this procedure three times and used the highest value.

After these preoperative evaluations, the patient was referred to the operating room and then to the ICU. During this period, no researcher had an influence on the procedures adopted by the team, and the patient managed based on other protocol.

After the patient's ICU stay, on their first day in the inpatient unit, they were divided into two groups [control group (CG) and training group (TG)]. Since the allocation of the groups was the admission order, the first patient was in the control group, the second in training, the third in control, the fourth in training, so on.

The CG received no specific intervention, being handled in accordance with the routine of the unit. However, the TG underwent evaluation of MIP and initiated inspiratory muscle training with a pressure linear load device (Threshold^®^ Respironics^®^ IMT), with 40% of the MIP, performing 3 sets with 10 repetitions. This training was performed twice daily until hospital discharge, according to the study protocol.

On the day of hospital discharge, patients in both groups were reevaluated in relation to the 6MWT and MIP in order to compare the results.

To evaluate the data we used the chi-square test for assessing the existence of association between the qualitative variables, the Student t test to analyze intergroup, the paired Student t test for intragroup analysis and Fisher's exact test in order to decrease the error associated with the chi-square test in small samples.

## RESULTS

From February to August 2015 were included 50 patients. In the TG, were allocated 11 (44%) men with a mean age of 56.4±13 years. In the CG, were included 16 (64%) men, with mean age of 57±14.7 years, admitted to the ICU of the Instituto Nobre de Cardiologia/Santa Casa de Misericordia in Feira de Santana/Bahia Brazil. Table 1 presents characteristics of patients included in the study.

**Table 1:** Clinical, demographic and surgical data for the group of patients undergoing cardiac surgery.

Variables	Training (n=25)	Control (n=25)	*P*
Gender			0.25^a^
Male	11	16	
Female	14	9	
Age (years)	56.4 ± 13	57 ± 14.7	0.86^b^
Surgery type			0.58^c^
CABG	18	19	
Valve	7	4	
Congenital	0	2	
Cardiopulmonary bypass time (min)	70.2 ± 19	68 ± 22	0.71^b^
MV time (hours)	7.4 ± 2.3	7.7 ± 3.1	0.72^b^
Length of stay (days)	6.2 ± 1.3	8.4 ± 2.8	0.002^b^

CABG=coronary artery bypass grafting; MV=mechanical ventilation

a Chi-square test;

b Student' t test;

c Fisher's exact test

In addition, in Table 1 we can see a statistically significant difference regarding the hospital stay between the groups [6.2±1.3 (GT) *vs.* 8.4±2.8 (GC) days], with *P*=0.002.

Other variables analyzed were the evolution of MIP among the groups and the distance covered in the walk test. Regarding the MIP in both groups, it declined significantly, from 97.5±18.2 to 69.5±14.9 (CG) and 94.2±16.2 to 83.1±19.1 (TG), but the TG had greater value at discharge compared to the CG in the same period 69.5±14.9 (CG) *vs.* 83.1±19.1 (GT), *P*=0.0073.

The values of 6MWD for the TG was significantly higher at discharge than CG [422.4±102.8 (GC) *vs.* 502.4±112.8, P=0.0031]. Furthermore, there was no difference in the distance covered by the TG between preoperative period and at discharge, suggesting maintenance of functional capacity. These data are shown in Table 2.

**Table 2:** Intra- and inter-group analysis of the mean maximal inspiratory pressure and distance walked in the 6MWD minutes of patients undergoing cardiac surgery.

	Preoperative Period	Discharge	*P*
MIP			
Control	97.5±18.2	69.5±14.9	0.00001
Training	94.2±16.2	83.1±19.1	<0.01
*P*	0.49	0.0073	
6MWD			
Control	499.6 ± 114.3	422.4 ± 102.8	0.0001
Training	516 ± 114.8	502.4 ± 112.8	<0.01
*P*	0.60	0.0031	

MIP=maximal inspiratory pressure; 6MWD=six-minute walk test Data presented as mean ± standard deviation. Student t test for paired intragroup analysis. Student's t-test for intergroup analysis.

## DISCUSSION

In our study we found a significant improvement in inspiratory muscle strength and functional capacity, through the 6MWT in patients undergoing cardiac surgery and exposed to an inspiratory muscle training program. In the literature, we could not find studies correlating inspiratory muscle training and physical functional performance in the postoperative period.

Iwama et al.^[11]^ concluded in their study with 134 patients that the 6MWT distance and predicted 6MWT variances were adequately explained by demographic and anthropometric attributes. This reference equation is probably most appropriate for evaluating the exercise capacity of Brazilian patients with chronic diseases.

Cardiac surgery with cardiopulmonary bypass determines systemic changes that culminate with worse outcomes after surgery. Among these systemic changes, it highlights the pulmonary origin. Systemic inflammatory response is characterized by the change in lung function ranging from reduced compliance, pulmonary edema and increased shunt fraction and reduced functional residual capacity^[12]^. This pulmonary dysfunction leads to increased work of breathing, which also is associated with impaired complacency of the chest wall^[13,14]^. The impaired complacency of the rib cage results in reduction of MIP.

In this study, all patients of the CG had reduced MIP at discharge. The TG showed similar MIP preoperatively and at discharge, as can be seen Table 2. Physical and functional performance were also higher in the group that underwent specific respiratory intervention, the opposite was observed by Carvalho et al.^[15]^. In that study, patients who received preoperative physiotherapy presented worse performance in the 6MWT than the CG.

Although not the focus of this study, evidence suggests that inspiratory muscle training is able to reduce postoperative complications such as pleural effusion and pneumonia. Hulzebos et al.^[16]^ found in a systematic review that besides reducing complications there is also shorter hospital stay time as observed in this study. However, this study is pioneer to state that the functional performance is improved in the group that took part of the inspiratory muscle training, culminating in a reduction of the hospital stay.

In a study published in 2009, the authors evaluated the impact of MIP on the hospital stay in the postoperative period of thoracic surgery, concluding that a MIP below 75% is predictor of an increased length of hospital stay^[17]^. Therefore, it should be given due attention to muscle dysfunction occurred in the postoperative period of cardiac and thoracic surgery. Consequently, inspiratory muscle training appears as an important strategy in reversing the muscle weakness. The muscle weakness presents sharp drop with inspiratory muscle training, consistent with the literature.

According to Cavenaghi et al.^[18]^, respiratory physiotherapy is an essential part in managing patients in the postoperative period, as it contributes significantly to a better prognosis of these patients can be carried out hygiene techniques, pulmonary reexpansion and muscle training.

Garcia & Costa^[19]^ evaluated 60 patients divided into three groups. The first group trained once a day, the second group trained twice a day and the third group did not participate in an respiratory muscle training. Most of the sample was male and the mean age was 59±9.6 corroborating with our results. Regarding the training, the authors concluded that this procedure is effective for the reestablishment of respiratory muscle function and maintaining airway patency.

Other studies analyze the impact of inspiratory muscle training insertion in the preoperative period. In a study performed by Ferreira et al.^[20]^, patients were trained for two weeks prior to cardiac surgery. They noted an improvement in forced vital capacity and maximum voluntary ventilation, but with no positive impact on the inspiratory and expiratory muscle strength. In a systematic review and meta analysis, Mans et al.^[21]^ evaluated the impact of training in 295 patients in the surgical preoperative showing that this technique improves muscle function without positive outcome on reducing the hospital stay.

Sobrinho et al.^[22]^ evaluated 70 patients and performing inspiratory muscle training in the same period of the previous research showing a reduction in hospital stay (8460 *vs.* 9970 minutes in patients who did not receive the pre-intervention training).

A training protocol was created using a linear pressure charging device twice a day with three sets of 10 repetitions and a charge of 40% of MIP. In this study there was significant recovery on the third day after surgery the MIP measures, however, no return to preoperative values, concluding that IMT is effective for recovery of tidal volume and vital capacity in patients undergoing revascularization^[23]^.

Nery et al.^[24]^ observed the presence of alterations in functional capacity in 179 patients undergoing coronary artery bypass grafting through the 6MWT. The patients were evaluated in two moments: before surgery and two years after. The average distance traveled by the group of active patients was 359±164.47 meters.

In another study, the authors aimed the 6MWT was able to evaluate the exercise capacity of patients after liver transplantation. In this research were evaluated thirteen patients in the seventh and fourteenth days after transplantation and found that the distance was about a hundred meters more on the fourteenth day, concluding that the 6MWT is useful, cheap and safe to assess exercise capacity in that patient profile^[25]^.

Pedrosa & Holanda^[26]^ evaluated 32 hypertensive and elderly women in order to compare different tests for aerobic endurance and functional mobility assessment. 6MWT, 2-minute step test (2MST) and the Timed up and go test (TUG) were compared, being verified a moderate positive correlation between the 6MWT and 2MST, thus, the authors conclude that the 6MWT can be replaced by 2MST in elderly with hypertension.

The present study has limitations that should be considered, such as impossibility to apply morbidity surveys in the preoperative period, since the admission had occurred one day before the surgery; lack of analysis of variables as risk score, body mass index and other comorbidities that can interfere with reduced muscle force or in the evolution of muscle strength during training. Although these limitations can affect these variables, the randomization process gave equal possibilities for insertion of patients in both groups.

## CONCLUSION

We can conclude that inspiratory muscle training protocol via a linear charging device generates increased MIP, which in turn was able to maximize the physical and functional performance in patients undergoing cardiac surgery.

New research should include more representative populations, assessing risk factors for muscle dysfunction and with different protocols to assess this outcome.

**Table:** 

Authors' roles & responsibilities	
ALLC	Conception and design study; analysis and/or data interpretation; manuscript redaction or critical review of its content; statistical analysis; final manuscript approval
TAM	Analysis and/or data interpretation; statistical analysis; final manuscript approval
DN	Conception and design study; realization of operations and/or trials; analysis and/or data interpretation; final manuscript approval
JL	Conception and design study; realization of operations and/or trials; analysis and/or data interpretation; final manuscript approval
MSE	Final manuscript approval
G F R A	Final manuscript approval
DLB	Statistical analysis; manuscript redaction or critical review of its content; final manuscript approval
JP	Conception and design study; analysis and/or data interpretation; statistical analysis; manuscript redaction or critical review of its content; final manuscript approval
